# Effect of total exemption from medical service co-payments on potentially inappropriate medication use among elderly ambulatory patients in a single center in Japan: a retrospective cross-sectional study

**DOI:** 10.1186/s13104-018-3320-y

**Published:** 2018-03-27

**Authors:** Junpei Komagamine, Kazuhiko Hagane

**Affiliations:** 1 Department of Internal Medicine, National Hospital Organization Tochigi Medical Center, 1-10-37, Nakatomatsuri, Utsunomiya, Tochigi 3208580 Japan; 2 Department of Pediatric Surgery, National Hospital Organization Tochigi Medical Center, 1-10-37, Nakatomatsuri, Utsunomiya, Tochigi 3208580 Japan

**Keywords:** Benzodiazepines, Drug costs, Potentially inappropriate medications, Social health insurance

## Abstract

**Objective:**

The effect of total exemption from medical service co-payments on drug prescribing practices has not been extensively evaluated. We conducted a retrospective cross-sectional study to evaluate the effect of total exemption from medical service co-payments on potentially inappropriate medication (PIM) and benzodiazepine use in elderly ambulatory patients. We defined PIM based on the Beers Criteria.

**Results:**

Six hundred seventy-one consecutive patients aged 65 years or older who routinely visited internal medicine physicians were included. Their mean age was 75.7 years, and 342 (51.0%) patients were men. The proportions of patients taking any PIMs or benzodiazepines were 37.7% and 16.2%, respectively. Of all patients, 62 (9.2%) were totally exempt from medical service co-payments. The patients who were totally exempt from medical service co-payments showed a significantly increased risk of PIM (OR 2.16, 95% CI 1.28–3.66) or benzodiazepine use (OR 2.12, 95% CI 1.16–3.87) compared with patients who were not. These associations did not change after adjusting for age, gender, comorbidities and polypharmacy. These findings should be confirmed in other settings or hospitals in Japan.

**Electronic supplementary material:**

The online version of this article (10.1186/s13104-018-3320-y) contains supplementary material, which is available to authorized users.

## Introduction

Potentially inappropriate medications (PIMs) are defined as medications that have an unfavorable balance of benefit and harm for many elderly adults [1]. Although several geriatric experts propose that PIMs, including benzodiazepines, should be avoided if possible in elderly patients [2–5], the prevalence of PIM use in elderly patients is high worldwide [6–9].

One of the risk factors for PIM use is a reduced out-of-pocket cost, which is covered by social health insurance systems [10, 11]. Although this decreased cost increases the use of essential medications, it also increases unnecessary or inappropriate medication use [10]. This moral conflict is problematic because inappropriate medications can result in excess costs for society and harmful outcomes for patients [12]. In Japan, since universal health coverage was established in 1961, approximately 3500 social health insurance plans have been established [13]. Under these insurance systems, some individuals are totally exempt from co-payments for medical services in Japan for several reasons [14]. For example, people on public assistance who cannot afford their prescriptions are exempted from these co-payments. These patients can receive all types of prescribed medications without paying the costs. However, as reduced out-of-pocket costs have been associated with an increased risk of PIM and benzodiazepine use [15, 16], total exemption from co-payments for medical services may lead to a greater increase in the use of these drugs among elderly patients. Considering the harmful effects of PIMs, including benzodiazepines [17], clarifying this relationship is important. Nonetheless, to our knowledge, no studies have evaluated this relationship in Japan. Therefore, our aim was to investigate whether total exemption from medical service co-payments, compared with other insurance plans, was associated with an increased risk of PIM and benzodiazepine use among elderly patients.

## Main text

### Methods

#### Study design and location

A retrospective cross-sectional study was conducted using the electronic medical records of the National Hospital Organization Tochigi Medical Center. This hospital is a 350-bed acute care community hospital and is one of five main hospitals that serve approximately 0.5 million individuals in Utsunomiya in the Tochigi prefecture in Japan.

#### Participants and inclusion criteria

By using the database of our hospital, we retrospectively screened all consecutive ambulatory patients aged 65 years or older who had appointments with internal medicine physicians between January 1, 2015, and January 31, 2015. We included only patients who had attended three or more visits to internal medicine physicians within a year before the index visit. Patients with missing data for medications prescribed from other hospitals were excluded. During the study period, 711 ambulatory patients who were at least 65 years old were identified. Forty patients were excluded due to a lack of data regarding medications prescribed from other hospitals. Therefore, a total of 671 patients were included in the final analysis. The mean patient age was 75.7 years, and 342 (51.0%) patients were men.

#### Exposure and comparison

Patients who were totally exempt from payment for any medications at the time of the index visit constituted the total exemption group, and the remaining patients were included in the control group. For example, patients receiving public assistance were included in the total exemption group, while patients supported by public insurance due to an intractable disease were classified as controls because they were exempted from co-payments only for specified disease-related medications [13, 14]. Among all the patients included in this study, 62 (9.2%) were included in the total exemption group. Of these patients, 60 (96.8%) were on public assistance, and one patient was on public insurance due to a law mandating assistance to Japanese orphans in China [14]. The remaining patient was totally exempt from payment for any medications for special reasons due to coverage provided by industrial accident compensation insurance [18]. Detailed information on the insurance plans of the patients included in the control group is shown in an Additional file 1: Table S1.

#### Data collection

Data were collected using the electronic medical records of the National Hospital Organization Tochigi Medical Center. Information on age, gender, social insurance, past medical history, Charlson comorbidity index (CCI) [19], and medications was retrieved from medical records at the time of the index visit. Medications included oral medications, inhalers, and injections, as well as as-needed medications. However, eye drops, intranasal infusers, over-the-counter drugs, and topical medications were excluded, as were medications that were indicated for apparent transient disease or were not administered at two or more consecutive visits. Data collection was performed from January 2017 to April 2017.

#### Outcome measures

The two primary outcomes were the proportions of patients taking at least one PIM or benzodiazepine. We defined PIMs based on the 2015 Beers Criteria of the American Geriatric Society [3]. We used two of the five components of the Beers Criteria: PIM use in older adults and PIM use in older adults due to drug-disease or drug-syndrome interactions, which may exacerbate the disease or syndrome. In Japan, few methods to evaluate the appropriateness of medications for elderly patients have been tested or validated. However, previous studies have found that the Beers Criteria may be applicable in the Japanese population [20, 21], and the Beers Criteria have been the most frequently used measure in Japanese research. Therefore, we selected the Beers Criteria. The secondary outcome was the proportion of patients taking at least one hypnotic drug. We compared the total exemption group with the control group regarding these outcomes.

### Statistical analysis

Assuming that 10% of all the patients are in the total exemption group and that the proportions of patients taking at least one PIM in the total exemption and control groups are 55% and 35%, respectively, a sample size of approximately 600 patients provides a power of 80% at a two-sided 5% significance level.

Demographic and other clinical characteristics of the study population were summarized using percentages or the mean and standard deviation (SD). The baseline characteristics of each group were compared using Fisher’s exact test for categorical variables and Student’s t test for continuous variables. We used Fisher’s exact test to evaluate the differences between each group in the proportion of patients taking any PIMs, benzodiazepines, or hypnotics. To evaluate whether total exemption from medical service co-payments independently affected PIM and benzodiazepine use, a multivariable analysis was conducted to examine the associations between the use of any PIMs and benzodiazepines and the following variables: age, gender, CCI, polypharmacy [8], and total exemption from medical service co-payments. Polypharmacy was defined as the use of five or more medications based on a past study [22] because no universal standard definition of polypharmacy is available. These analyses were conducted from April 2017 to February 2018 using IBM SPSS Statistics Base version 21.0 (IBM corporation, Nihonbashi, Tokyo, Japan) or Excel statistical software package version 2.11 (Bellcurve for Excel; Social Survey Research Information Co., Ltd., Tokyo, Japan), and the level of statistical significance was *p* < 0.05.

### Results

The baseline characteristics of the patients are presented in Table 1. Of the 671 patients, the mean CCI was 1.9, and the mean number of total medications was 5.0. Compared with the controls, the patients in the total exemption group were significantly younger and were more often men and current smokers. The total exemption group also had significantly higher CCI scores and a higher number of total medications.

**Table 1:** Baseline characteristics of the 671 elderly ambulatory patients

Characteristics	Total N = 671	Total exemption N = 62	Control N = 609	*p* value^d^
Age, mean ± SD	75.7 ± 7.5	71.3 ± 6.0	76.1 ± 7.5	< 0.001
Men, n (%)	342 (51.0)	42 (67.7)	300 (49.3)	0.01
Women, n (%)	329 (49.0)	20 (32.3)	309 (50.7)	0.01
CCI, mean ± SD	1.9 ± 1.7	2.4 ± 2.1	1.9 ± 1.7	0.04
Current smoker,^a^ n (%)	77 (14.0)	25 (43.1)	52 (10.5)	< 0.001
Regular drinker,^b^ n (%)	128 (23.8)	18 (31.0)	110 (23.0)	0.19
Number of prescribers, mean ± SD	1.4 ± 0.7	1.6 ± 0.8	1.4 ± 0.7	0.08
Number of medications				
Total, mean ± SD	5.0 ± 3.0	6.2 ± 3.6	4.9 ± 2.9	0.01
Five or more medications, n (%)	339 (50.5)	37 (59.7)	302 (49.6)	0.14
Past medical history, n (%)				
Myocardial infarction	40 (6.0)	3 (4.8)	37 (6.1)	1.00
Heart failure	50 (7.5)	4 (6.5)	46 (7.6)	1.00
Angina	58 (8.6)	8 (12.9)	50 (8.2)	0.23
Atrial fibrillation	80 (11.9)	6 (9.7)	74 (12.2)	0.68
Ischemic stroke	90 (13.4)	11 (17.7)	79 (13.0)	0.33
Hemorrhagic stroke^c^	12 (1.9)	0 (0.0)	13 (2.1)	0.62
Peptic ulcer	126 (18.8)	17 (27.4)	109 (17.9)	0.09
GERD	108 (16.1)	11 (17.7)	97 (15.9)	0.72
NIDDM	212 (31.6)	25 (40.3)	187 (30.7)	0.15
IDDM	18 (2.7)	0 (0.0)	18 (3.0)	0.40
Hypertension	483 (72.0)	41 (66.1)	442 (72.6)	0.30
Dyslipidemia	360 (53.7)	25 (40.3)	335 (50.0)	0.03
Chronic kidney disease	234 (34.9)	14 (22.6)	220 (36.1)	0.04
Rheumatic disease	34 (5.1)	4 (6.5)	30 (4.9)	0.54
Asthma or COPD	81 (12.1)	12 (19.4)	69 (11.3)	0.10
Dementia	43 (6.4)	2 (3.2)	41 (6.7)	0.42
Active cancer	31 (4.6)	6 (9.7)	25 (4.1)	0.06
Depression	34 (5.1)	4 (6.5)	30 (4.9)	0.54
Osteoporosis	95 (14.2)	9 (14.5)	86 (14.1)	0.85

^a^Among 551 patients (58 patients in the total exemption group and 493 patients in the control group)

^b^Among 537 patients (58 patients in the total exemption group and 479 patients in the control group)

^c^Hemorrhagic stroke included cerebral hemorrhage and subarachnoid hemorrhage

^d^Comparison between the total exemption and control groups was performed using Fisher’s exact test for categorical variables and Student’s t test for continuous variables

Table 2 shows the prevalence of PIM and benzodiazepine use in each group. Of the entire sample, the proportions of patients taking any PIMs or benzodiazepines were 37.7% and 16.2%, respectively. The proportion of patients taking any PIMs was significantly higher in the total exemption group than that in the control group (54.8% and 36.0%, respectively). The proportion of patients taking benzodiazepines was also significantly higher in the total exemption group than that in the control group (27.4% and 15.1%, respectively). However, the proportion of patients taking hypnotics did not significantly differ between the two groups, although the total exemption group tended to have more frequent hypnotic use than the control group.

**Table 2:** Prevalence and characteristics of potentially inappropriate medication^a^ use among the 671 elderly ambulatory patients

	Total N = 671	Total exemption N = 62	Control N = 609	*p* value^c^
Number of PIMs, mean ± SD	0.5 ± 0.8	0.9 ± 0.9	0.5 ± 0.8	0.002
Any PIMs, n (%)	253 (37.7)	34 (54.8)	219 (36.0)	0.01
Category of PIM, n (%)				
Benzodiazepines	109 (16.2)	17 (27.4)	92 (15.1)	0.02
Proton-pump inhibitors	87 (13.0)	11 (17.7)	76 (12.5)	0.24
Hypnotics^b^	33 (4.9)	5 (8.1)	28 (4.6)	0.22
NSAIDs	25 (3.7)	4 (6.5)	21 (3.4)	0.28
Peripheral alpha-1 blockers	24 (3.6)	3 (4.8)	21 (3.4)	0.48
Antidepressants	14 (2.1)	2 (3.2)	13 (2.1)	0.64
Digoxin	7 (1.0)	2 (3.2)	5 (0.8)	0.13
Antipsychotics	7 (1.0)	0 (0.0)	7 (1.1)	1.00
Ticlopidine or dipyridamole	6 (0.9)	1 (1.6)	5 (0.8)	0.44
First-generation antihistamines	4 (0.6)	0 (0.0)	4 (0.7)	1.00
Others	26 (3.9)	3 (4.8)	23 (3.8)	0.73

^a^PIM was defined based on the 2015 American Geriatric Society Beers Criteria

^b^Non-benzodiazepine and benzodiazepine receptor agonist hypnotics

^c^Comparison between the total exemption and control groups was performed using Fisher’s exact test for categorical variables and Student’s t-test for continuous variables

Table 3 shows the results of the multivariable logistic regression analysis performed to determine the predictive factors of the use of any PIMs or benzodiazepines among elderly ambulatory patients. Total exemption from medical service co-payments and polypharmacy were independently associated with a higher risk of any PIM or benzodiazepine use. Increasing age was significantly associated with a higher risk of any PIM use, but not benzodiazepine use. Neither the CCI nor gender were independent predictive factors of any PIM or benzodiazepine use.

**Table 3:** Summary of the multivariable logistic regression results to predict the use of any PIMs^a^ or benzodiazepines among the 671 elderly ambulatory patients

	Odds ratio (95% CI)^b^	
	Unadjusted	Adjusted^c^
PIM use		
Age	1.05 (1.02–1.07)**	1.03 (1.00–1.06)*
Women	1.26 (0.92–1.72)	1.44 (0.99–2.08)
CCI	1.12 (1.02–1.22)*	0.96 (0.86–1.07)
Polypharmacy^d^	8.12 (5.62–11.75)**	8.02 (5.44–11.8)**
Total exemption from co-payments	2.16 (1.28–3.66)*	2.72 (1.45–5.08)*
Benzodiazepine use		
Age	1.03 (1.00–1.06)*	1.02 (0.99–1.05)
Women	1.07 (0.71–1.61)	1.07 (0.69–1.67)
CCI	1.01 (0.90–1.14)	0.88 (0.77–1.01)
Polypharmacy^d^	4.86 (2.96–7.99)**	4.97 (2.97–8.32)**
Total exemption from co-payments	2.12 (1.16–3.87)*	2.38 (1.22–4.61)*

^a^PIMs were defined based on the 2015 American Geriatric Society Beers Criteria

^b^The level of statistical significance was set at *p* < 0.05. Asterisks indicate a significant association between the selected variable and the use of PIMs and benzodiazepines; *p < 0.05, **p < 0.001

^c^These variables were adjusted for age, gender, Charlson comorbidity index, polypharmacy, and total exemption from medical service co-payments

^d^Polypharmacy was defined as the use of five or more medications

### Discussion

The results of this study showed that compared with other forms of social health insurance, total exemption from medical service co-payments was associated with a significantly increased risk of the use of PIMs and benzodiazepines among elderly ambulatory patients, although no significant difference was found between the groups regarding the use of hypnotics. To our knowledge, this is the first study to show an increased risk of PIM and benzodiazepine use among elderly patients due to total exemption from medical service co-payments for medical services in Japan.

Our findings are consistent with those of previous studies showing that a reduced out-of-pocket cost increased the use of PIMs [10, 11], although the extent of the effect in the present study seems larger than that in past studies. Given that the extent of the effect observed in this study is similar to that in past studies, indicating that completely excluding benzodiazepines from coverage significantly reduced benzodiazepine use in elderly patients [15, 16], total exemption from co-payments may have a greater effect on the increased risk of PIM and benzodiazepine use compared with partial exemption from co-payments. Considering the harmful effects of PIMs [17, 23] and benzodiazepines [24–27], a strategy to prevent and reduce their use among elderly patients who are totally exempt from co-payments is needed.

In this study, total exemption from co-payments was not statistically associated with an increased risk of hypnotic use. However, given that the number of patients taking hypnotics was small in this study, further studies are warranted to evaluate this association.

### Conclusions

Total exemption from co-payments for medical services was significantly associated with an increased risk of PIM and benzodiazepine use among elderly ambulatory patients. Strategies to prevent increased use of PIMs while protecting free access to medical services are needed.

## Limitations

Our results should be interpreted in the context of several limitations. First, the study used a retrospective cross-sectional design. Second, we excluded patients who had attended fewer than three visits to internal medicine physicians within a year before the index visit, thus introducing a selection bias. Third, we did not evaluate the severity of comorbidities, which may also affect the risk of PIM and benzodiazepine use in the total exemption group. Fourth, this study was limited to a single center and a small sample size; consequently, the results cannot be easily generalized to other populations. Therefore, these findings should be confirmed by conducting a population-level study in the future. Fifth, the control group in this study included very heterogeneous patients who were covered by several social health insurance plans in Japan. However, due to the complexity of the Japanese social health insurance system [13], dividing the control group further according to their required co-payments for medications was difficult. Sixth, our assessment did not include patients taking essential medications [10]. Finally, we did not evaluate associations between PIM and benzodiazepine use and clinically important outcomes such as mortality and adverse drug events.

## Additional file

**Additional file 1: Table S1.** The proportions of patients under various insurance plans according to tiers^a^ in the 609 elderly ambulatory patients included in the control group. ^a^Insurance plans were classified into four tiers based on a past article (Lancet 2011;378:1106-15). ^b^Elderly patients were covered by one of these insurance plans unless they received public assistance. Under these insurance plans, adult patients aged less than 75 years must pay 30% of medical costs. Patients aged 75 years and older only pay 10% of medical costs. However, co-payment cost is also affected by several factors, such as enrollees’ income, monthly co-payment threshold, and combination with other insurance plans.

## Abbreviations

CCI: Charlson comorbidity index
CI: confidence interval
COPD: chronic obstructive pulmonary disease
GERD: gastroesophageal reflux disease
IDDM: insulin-dependent diabetes mellitus
NIDDM: non-insulin-dependent diabetes mellitus
NSAIDs: non-steroidal anti-inflammatory drugs
OR: odds ratio
PIM: potentially inappropriate medication
SD: standard deviation
