# Supplementary material for: Effect of total exemption from medical service co-payments on potentially inappropriate medication use among elderly ambulatory patients in a single center in Japan: a retrospective cross-sectional study
Source: BMC Res Notes. 2018 Mar 27;11:199. doi: 10.1186/s13104-018-3320-y (PMC5870246; doi:10.1186/s13104-018-3320-y)
Supplement: Supplementary file 1 — Additional file 1: Table S1. The proportions of patients under various insurance plans according to tiersa in the 609 elderly ambulatory patients included in the control group. aInsurance plans were classified into four tiers based on a past article (Lancet 2011;378:1106-15). bElderly patients were covered by one of these insurance plans unless they received public assistance. Under these insurance plans, adult patients aged less than 75 years must pay 30% of medical costs. Patients aged 75 years and older only pay 10% of medical costs. However, co-payment cost is also affected by several factors, such as enrollees’ income, monthly co-payment threshold, and combination with other insurance plans. [file 13104_2018_3320_MOESM1_ESM.docx]

**Table S1.** The proportions of patients under various insurance plans according to tiers^a^ in the 609 elderly ambulatory patients included in the control group.

| **Insurance plans^b^** | **Total**  **N = 609** |
| --- | --- |
| **First tier**  Society-managed Health Insurance “Kumiai kenko hoken”  Mutual aid association “Kyosai kumiai” | 10 (1.6)  1 (0.2) |
| **Second tier**  Association-Kempo Health Insurance “Kyokai Kempo” | 30 (4.9) |
| **Third tier**  National Health Insurance “Kokumin kenko hoken” | 224 (36.8) |
| **Fourth tier**  Later Elders’ Health Insurance “Koki koreisha iryo seido” | 344 (56.5) |

^a^Insurance plans were classified into four tiers based on a past article (Lancet 2011;378:1106-15).

^b^Elderly patients were covered by one of these insurance plans unless they received public assistance. Under these insurance plans, adult patients aged less than 75 years must pay 30% of medical costs. Patients aged 75 years and older only pay 10% of medical costs. However, co-payment cost is also affected by several factors, such as enrollees’ income, monthly co-payment threshold, and combination with other insurance plans.
